# The Case against the Voluntary Hospital

**Published:** 1917-06-09

**Authors:** 


### THE EDITOR'S LETTER-BOX.

### The Case Against the Voluntary Hospital.

*To the Editor of* The Hospital.

Sir,—It is significant of the increasing interest of the public in matters touching the health of the nation that the *Daily Telegraph* should, in these days of paper shortage, have devoted close upon three columns last week to the discussion of a scheme for altering the present system of allotting hospital appointments. The author, Mr. E. W. Morris, claims that the members of the medical staff of more than one hospital in London are among his most valued personal friends, so that his criticisms and strictures should at least have the merit of being impartial. He claims, with a certain measure of justice, that the present method of filling the vacancies upon the staff of one of the teaching hospitals involves so long a period of waiting that only those with considerable private means can afford to "hang on," filling poorly paid posts, till their time comes, and that thus many potential surgeons and clinicians are by stress of circumstances forced to take up general practice from financial reasons. This is no doubt true enough.

But some in touch with hospital life may not agree with the following : "It is not by any means certain that his [the future consultant's] years of waiting for his vacancy have fitted him for a consultant post—he has been a registrar, or a demonstrator in anatomy or in physiology, or an assistant in a laboratory. Does the system make for the best man to be elected to the hospital staff? He is elected by the governors probably on the recommendation of his future colleagues on the staff. Are his teaching abilities always taken into account, or his power of investigation and research, or his organising ability for the management of a clinique? . . . Are conditions ever imposed upon a man by his future colleagues as a condition of their support, such as, for instance, a limitation of the operations he may perform? In deciding on the man to be recommended, is his ability taken into consideration absolutely without reference to his danger as a future rival to the already existing staff?"

Petty jealousies there doubtless are—where, indeed, are there not?—but such tacit agreements as are here suggested certainly strike a new note in hospital administration. Surely the writer must know that in the case of an assistant surgeon—or a full surgeon, for the matter of that—the number of operations performed depends solely upon the number of beds allocated to them—a fixed and, in pre-war days, invariable quantity. That any agreement as to not performing a certain operation, claimed, we take it, to be the monopoly of a surgeon already on the staff, would be as absurd as it would be impracticable, is evident, since every surgeon has a free hand in admitting the cases he wishes to his own beds, and no operation can ever be claimed as the exclusive monopoly of any surgeon.

The years of waiting, spent, according to Mr. Morris, in humdrum drudgery, are, on the contrary, to those who can afford them, a time of the most valuable opportunities for observation and research. What better proof of a man's teaching abilities than that he has spent a year, or it may be two, as medical or surgical registrar, demonstrating cases at the bedside to students? What better stimulus to accurate diagnosis than the daily verification of his findings on the operating table and in the *post-mortem* room, the knowledge that his observations are thus put to the test, and any error carefully noted by the students whom it is his business to teach?

Has he never heard of the appointments of resident surgical officer, resident obstetrician, etc., where the future surgeon or gynæcologist has priceless opportunities for perfecting his skill?

That the embryo teacher and consultant should spend a portion of his time lecturing upon anatomy or physiology, or in laboratory work, would seem to afford that very proof of his didactic powers and his enthusiasm for research, which, it is urged, are not taken sufficiently into consideration in filling staff appointments. Moreover, the surgeon can hardly be too familiar with the structure of the human frame, or the physician with its functions in the healthy state; and though the great anatomist is not of necessity the brilliant surgeon, or the professor of physiology a profound clinician, the process of selection is too rigorous for mere academic attainments to counterbalance practical achievement.

But in dealing with the training of the rank and file of the medical profession Mr. Morris is on surer ground. Owing to the comparatively small number of teaching hospitals, and the small numbers of surgeons and physicians on their staffs, less than half the men who qualify are in normal times able to hold a post-graduate appointment at the hospital where they received their training. If he is lucky enough to obtain a house-surgeoncy or physicianship, he becomes "a good doctor for the rest of his life, if there is any of the healing instinct in him at all. . . . Every man who passes his qualifying examination should by right hold a hospital appointment in his hospital for some period or another, and should enjoy the benefit of taking responsibility under guidance and check; it should be part of his training to do so, and it could be done *if the honorary staff were doubled and the number of beds allotted to each reduced.*"

But our critic goes further than this; he urges the abolition of the honorary principle, root and branch. The Harley Street consultant is, he maintains, too busy for investigation or research, relying more and more on the bacteriologist, the clinical microscopist, the chemical pathologist. He tends more and more to become simply the able technician who utilises the tools placed by others ready to his hand. As an alternative the hospitals would be staffed by men holding whole- or part-time appointments, paid at a fixed scale of remuneration dependent upon the number of days spent per week; ". . . men who would be willing to give their whole time, say, for five years to the work of the hospital for a fixed salary. Then let such a man gradually have his hospital time reduced, as he gets wiser and wiser, and his hospital salary proportionately reduced, thereby enabling him gradually to slide into private practice. New men would come on as 'whole-timers'; they, too, gradually working up to reduced salary and reduced hours of hospital duty. Thus many more men would have the great educational advantage of being on a hospital staff for a period, not an honorary staff, but a well-paid staff."

Each man, or rather a group of men, working under a senior, who would naturally be the worst paid, would have twenty or thirty beds set apart for the investigation of a particular disease, in whose nature and treatment they would be expected to specialise.

As to the cost, it would, Mr Morris naively remarks, be paid by the State. "There must be a State Department of Health guiding and influencing all existing departments dealing with the health of the people. Such

☛ Continued on page iv.

